# Seasonality of diet costs reveals food system performance in East Africa

**DOI:** 10.1126/sciadv.abc2162

**Published:** 2020-12-04

**Authors:** Yan Bai, Elena N. Naumova, William A. Masters

**Affiliations:** Friedman School of Nutrition Science and Policy, Tufts University, Boston, MA 02111, USA.

## Abstract

Seasonal fluctuations in food prices reflect interactions between climate and society, measuring the degree to which predictable patterns of crop growth and harvest are offset by storage and trade. Previous research on seasonality in food systems has focused on specific commodities. This study accounts for substitution between items to meet nutritional needs, computing seasonal variation in local food environments using monthly retail prices for 191 items across Ethiopia, Malawi, and Tanzania from 2002 through 2016. We computed over 25,000 least-cost diets meeting nutrient requirements at each market every month and then measured the magnitude and timing of seasonality in diet costs. We found significant intensity in Malawi, Tanzania, and Ethiopia (10.0, 6.3, and 4.0%, respectively), driven primarily by synchronized price rises for nutrient-dense foods. Results provide a metric to map nutritional security, pointing to opportunities for more targeted investments to improve the year-round delivery of nutrients.

## Teaser

Synchronized food retail prices raise seasonal peaks in the cost of nutritious diets, revealing new ways to improve food systems.

## INTRODUCTION

High food prices limit consumption and harm well-being for low-income people (*1*–*3*). This study addresses the predictable component of price fluctuations, focusing on recurring seasonal peaks of consistent timing and intensity (*4*). All kinds of food price volatility may affect nutrition and health (*5*–*8*), but seasonality is of particular interest because it measures the degree to which people have improved agriculture and food systems sufficiently to overcome predictable climate fluctuations. Improvements in storage and transport have helped stabilize prices over time (*9*, *10*), but there remains significant seasonality in wholesale prices at many market locations in Africa (*11*). This study measures seasonal variation in retail prices across all food groups and diet costs in a way that allows substitution among items to meet nutrient needs.

**Fig. 1:**
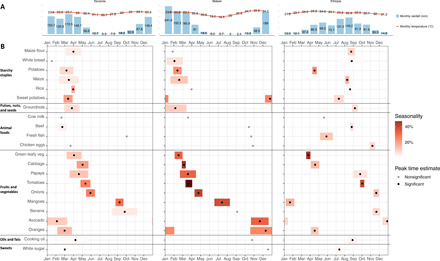
Intensity and timing of seasonality in market prices for commonly consumed foods in Tanzania, Malawi, and Ethiopia. (**A**) National average monthly rainfall (in millimeters) and temperature (in degrees Celsius) between 1991 and 2016 (*32*). (**B**) Ninety-five percent confidence intervals (CIs) around the peak month for each food, shown as a black dot, with the magnitude of intensity shown by the color gradation of each bar. Gray dots show the peak month for foods without statistically significant harmonic seasonality. Price variation is estimated from data in local currency units (LCUs) per item, on average over all market locations in each country shown.

Our study uses government file data on monthly retail prices and harmonic regression analysis to measure the timing and intensity of seasonality in three East African countries, Tanzania, Malawi, and Ethiopia, chosen because of their vulnerability to malnutrition and also variation in geography north and south of the equator, as well as variation in altitude and distance from ocean ports or land transport routes. The inclusion of these three countries is also due to availability of relatively high quality of food price data. From Tanzania, we have prices for 61 foods at 21 market locations from 2011 through 2015; in Malawi, we have 48 foods at 29 markets from 2007 through 2016; and in Ethiopia, prices are for 82 foods at 120 markets from 2002 through 2016. The total number of market-month observations is 3480 in Malawi, 1236 in Tanzania, and 20,806 in Ethiopia. Our harmonic model uses sine and cosine functions to estimate smooth, symmetric fluctuations of each item’s price or diet costs over time, in this case, with one cycle each year reflecting the region’s unimodal rainfall (Fig. 1A). The seasonal intensity of price variation is the difference between its annual peak and nadir normalized to a unit-free percentage of the nadir. This approach allows us to measure the magnitude and timing of peaks for different combinations of foods at different locations, using 95% confidence intervals (CIs) around the estimated intensity to test for statistical significance.

The prices we use were originally collected to measure inflation for each country’s consumer price index and are repurposed here to track the least-cost sources of 21 essential nutrients and dietary energy in the proportions needed for an active and healthy adult woman. We and others focus on diet costs for women of reproductive age because they are often at risk of malnutrition, with severe consequences for themselves and for child health (*12*). To allow for substitution among foods in delivering nutrients, we computed the least-cost combination of foods at each place and time needed to meet all requirements, and compared that to bare subsistence cost of daily energy from starchy staples only (*13*, *14*). Each food list includes a wide variety of nutrient sources, including starchy staples, pulses/nuts/seeds, animal foods, fruits/vegetables, oils/fats, and sweets. Not all foods are available at each market every month, but only 102 of the 25,522 market-months in our study had an insufficient variety of foods to meet all nutrient needs, and all of those were in Ethiopia. After computing least-cost diets, we used harmonic regression to extract the seasonal component of variation in cost of nutrients and daily energy at each location, and report differences in timing and intensity as a metric of food system performance and vulnerability to climatic fluctuations. Our method would also be useful to identify price anomalies due to disruptions such as armed conflict or disease outbreaks.

Measuring seasonality in the cost of nutrients over all major food groups, allowing for substitution among items as their relative prices change, allows us to compare the ability of local farmers and traders to deliver year-round access to all essential nutrients in the proportions needed by people. This permits us to quantify the nutritional performance of local agroecosystems, distinguishing nutrition security from food security, and identify how each type of food contributes to seasonal variation so as to guide interventions that could improve year-round access to a nutritious diet.

## RESULTS

### Seasonality of individual food prices

The timing of harvest leads to seasonality in prices at each market location, if not offset by storage and trade with other places. Figure 1A reveals the national average pattern in rainfall and temperature over each calendar year. Tanzania and Malawi have a cooler dry season approximately from May to October, and Ethiopia, located north of the equator, has a dry season from November to March. We found that these recurring cycles lead to statistically significant seasonality in most food items in all three countries (36 of 61 items in Tanzania, 31 of 48 items in Malawi, and 72 of 82 items in Ethiopia; tables S1 to S3).

To visualize these data in Fig. 1B, we show the estimated seasonal intensity and peak timing for 22 standard items from six major food groups. Fruits and vegetables generally have stronger seasonality than other food groups, especially in Malawi. For example, tomatoes have a high seasonal intensity of 25.8% (18.7%, 33.3%) in Tanzania, 60.3% (46.1%, 75.9%) in Malawi, and 38.7% (31.7%, 46.2%) in Ethiopia. High seasonal intensities were also found in prices of locally representative dark leafy vegetables, notably 12.8% (7.5%, 18.4%) for mchicha (amaranth leaves) in Tanzania, 32.7% (22.2%, 44.2%) for rape leaves and 20.7% (10.7%, 31.6%) for pumpkin leaves in Malawi, and 46.9% (38.0%, 56.4%) for kale in Ethiopia. Potatoes and sweet potatoes also have high seasonality in their prices, while cereal grains and pulses, nuts, and seeds have less seasonal fluctuation, and animal-sourced foods have little or no seasonality in these data. Seasonal peaks in Tanzania and Malawi were synchronized for starchy staples and pulses/nuts/seeds in the late rainy seasons before harvesting, while fruits and vegetables have diverse price peaks that could help to stabilize diet costs if they offer similar nutrients, allowing substitution among them over the course of each year.

### Seasonality of diet costs

The ability of local food systems to deliver all nutrients needed for health is revealed by the cost of nutrient adequacy from all foods, which we abbreviate CoNA. We compare that to the cost of caloric adequacy from starchy staples, abbreviated CoCA, which is what would be needed for bare subsistence at each location every month. National average levels of CoNA over the period of observation were TZS (Tanzanian shilling) 912.1 [$1.50 in 2011 USD (U.S. dollar) at purchasing power parity (PPP) prices] in Tanzania, MWK (Malawian kwacha) 129.6 ($1.21) in Malawi, and ETB (Ethiopian birr) 6.74 ($1.34) in Ethiopia. These costs were 2.41, 3.11, and 3.49 times the country’s average level of CoCA required for subsistence (tables S4 to S6).

**Fig. 2:**
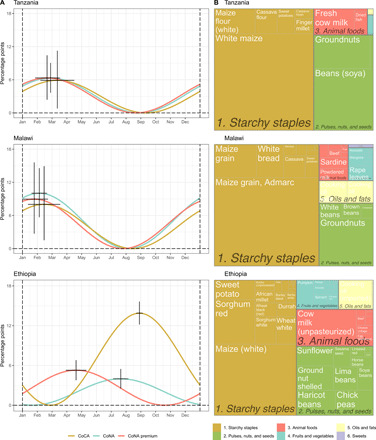
Seasonality in diet costs and composition of least-cost nutrient adequate diets in East Africa. (**A**) Estimated harmonic seasonality over a 1-year cycle for the three indicators, with error bars showing 95% CIs around the magnitude of seasonal intensity along the vertical axis and peak month along the horizontal axis. (**B**) Average energy composition by food group and item of the least-cost diet selected for CoNA over all observations in each country. CoCA is a least-cost diet that meets energy needs using only starchy staples. The CoNA premium is the cost of meeting nutrient requirements beyond daily energy, defined as CoNA-CoCA, in LCUs per day.

Seasonal fluctuation in the overall cost of all nutrients is large and statistically significant. As shown in Fig. 2A and tables S4 to S6, seasonality was much stronger in Malawi with a seasonal intensity of 10.0% (5.7%, 14.6%), compared to 6.3% (3.7%, 9.0%) in Tanzania and 4.0% (2.5%, 5.5%) in Ethiopia. Seasonal intensities in CoCA were significant in all three countries. The intensity was strongest at 13.9% (12.2%, 15.6%) in Ethiopia, and 8.0% (1.5%, 14.9%) in Malawi and 5.9% (0.8%, 11.3%) in Tanzania. The premium for nutrients above dietary energy, measured by the gap between CoNA and CoCA, also has significant seasonality with an intensity of 6.3% (2.4%, 10.4%) in Tanzania, 9.0% (2.7%, 15.6%) in Malawi, and 5.3% (3.8%, 6.8%) in Ethiopia.

**Fig. 3:**
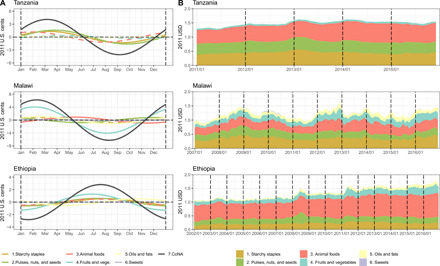
Seasonality in diet costs by food group over time. (**A**) Estimated harmonic seasonality over a 1-year cycle for the overall CoNA and for the selected components of that diet from each of the six food groups. Dashed lines are not statistically significantly different from zero. (**B**) Contribution of each food group to the CoNA each month, averaged over all marketplaces in the country shown. Diet costs are converted to USD at PPP exchange rates.

**Fig. 4:**
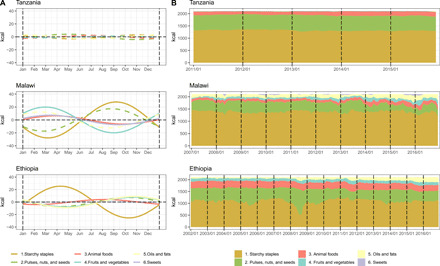
Seasonality in the composition of least-cost diets by food group over time (in kilocalories per day). (**A**) Predicted seasonal curves over a year cycle of energy intakes in kilocalories from six food groups; the dashed line means insignificant result. (**B**) Average energy compositions of CoNA over markets contributed by six food groups. The total daily energy intake is 2107.6 kcal, required by a woman between 19 and 30 years old under low active physical activity level with a height of 163 cm and a weight of 57 kg.

Peak timings of the three indicators in Malawi and Tanzania were estimated to be about 3 months before the harvest season starting in May. In Ethiopia, although CoCA was estimated to peak in late August, which is about 2 months before the start of harvest season in November, CoNA and CoNA premium peaked earlier in late July and mid-April, respectively. The timing and magnitude of these peaks reflect the limited degree to which different foods can substitute for each other to deliver all required nutrients around the year. As shown in Fig. 3A, the cost of each food group in a least-cost diet varies over time, with high levels of overall seasonality in Malawi driven by its seasonality in fruit and vegetable prices. A different view of these substitutions is presented in Fig. 4, as each food group’s contribution of total calories, which has significant seasonality in Malawi and Ethiopia but not in Tanzania. In Malawi, energy intake from starchy staples in CoNA becomes minimum before the harvest season and, therefore, more energy from fruits and vegetables, animal foods, and sweets. Figure 3B also reveals time trends in CoNA, for which the national averages increased from $1.31 to $1.56 over the 2011–2015 period in Tanzania, from $0.96 to $1.46 over the 2007–2016 period in Malawi, and from $1.04 to $1.68 over the 2002–2016 period in Ethiopia. In both Malawi and Tanzania, seasonality in the cost of fruits and vegetables contributed the most in the seasonality of CoNA (Fig. 3A), although fruits and vegetables do not take a large portion in total cost or energy of CoNA (Figs. 2B and 4B and tables S4 to S6).

**Fig. 5:**
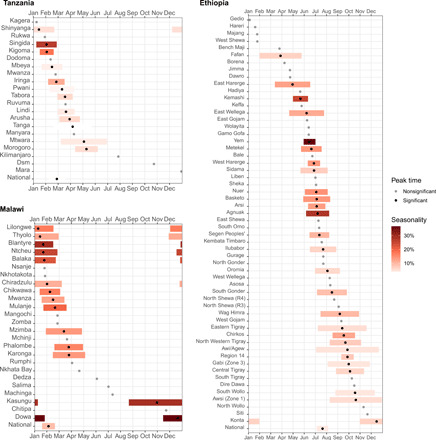
Intensity and timing of seasonality in diet costs across 21 regions of Tanzania, 25 districts of Malawi, and 57 zones of Ethiopia. Data shown are 95% CIs around the peak month in each location, shown as a black dot, with the magnitude of intensity shown by the color gradation of each bar. Gray dots show the peak month in locations without statistically significant seasonality in diet costs, as measured by the CoNA.

**Fig. 6:**
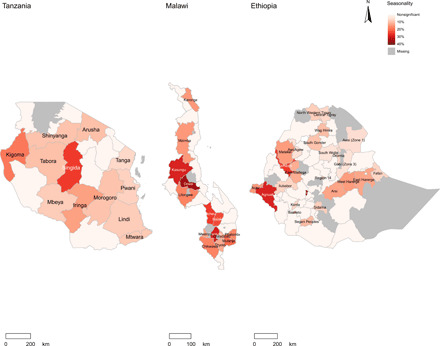
Geographical distribution of seasonality in diet costs within Tanzania, Malawi, and Ethiopia. Color gradations show the magnitude of estimated seasonal intensity in the CoNA.

Seasonal intensity in CoNA also presents great regional variations within countries. Regional results are shown in Figs. 5 and 6, where 12 of 21 regions in Tanzania, 14 of 25 districts in Malawi, and 27 of 57 zones in Ethiopia showed significant results. In Tanzania, the inland region of Singida and the west border region of Kigoma showed strong seasonality in CoNA with an intensity of 24.7% (8.2%, 43.7%) and 18.2% (9.9%, 27.2%). In Malawi, five districts suffered severe seasonality with an intensity of more than 20%, among which the Dowa district, close to the capital of Lilongwe City, showed a seasonal intensity of 35.2% (15.5%, 58.2%), and its peak timing was estimated approximately 1 month earlier than the national estimation. In Ethiopia, three zones had unusual higher seasonality in CoNA than the rest of the country, which are Kemashi with an intensity of 25.2% (16.9%, 34.1%) and Agnuak with an intensity of 27.7% (3.4%, 57.8%) on the west borders to Sudan and South Sudan, as well as Yem, a special woreda in the Southern Nations, Nationalities, and Peoples’ Region, with a seasonal intensity of 36.7% (22.7%, 52.3%). Last, we note the role of variation in individual dietary requirements, which affects the level of cost but has little effect on seasonality. For example, a higher level of physical activity would require 12% more daily energy, which raised CoNA by about 4% but led to negligible differences in the timing or intensity of seasonality.

## DISCUSSION

This paper introduced a combination of techniques to characterize spatiotemporal variation in food prices across three countries in East Africa, measuring the ability of local farmers and traders to achieve year-round delivery of all essential nutrients at low cost despite climatic fluctuations. We used government file data on a total of 191 items at 170 locations in various years from 2002 through 2016, solved for the least-cost combination of foods needed to meet requirements for 21 essential nutrients and dietary energy at each of 25,522 market-months, and then applied harmonic regression to estimate seasonal intensity and peak timing of diet costs at each location. Three important findings were found:

First, most individual foods have significant seasonality in retail prices, extending previous observations about major commodities to all food categories. Fruits and vegetables have the largest seasonal price variations, which averages over 20% for 7 of 21 items in Tanzania, 14 of 17 items in Malawi, and 8 of 24 items in Ethiopia. Items such as carrots, mangoes, papaya, oranges, avocado, tomatoes, green peppers, and onions are important not only for the essential nutrients they provide but also for other aspects of diet quality and local livelihoods. Foods that are more easily stored and transported, such as cereal grains and pulses, nuts, or seeds, have lower levels of seasonality than the highly perishable fruits and vegetables. We also find that seasonality in the prices of widely traded grains is lower on retail markets than previous studies had found in wholesale prices on commodity markets (*11*), implying that wholesale-to-retail margins help stabilize consumer prices. Nonetheless, peak times for various food groups tend to be synchronized before harvests in all three countries, limiting year-round access to all essential nutrients.

Next, even after allowing for substitution among foods, overall diet costs using the least-cost sources of nutrients and energy fluctuate seasonally in ways that are statistically and nutritionally significant. Substitution away from fruits and vegetables worsens diet quality during the lean season (*15*), and we find that scarcity of nutrient-dense foods typically precedes scarcity of calories from starchy staples as the peak timing for CoNA is earlier than the peak for CoCA. We also find large regional variation in the seasonality of diet costs, revealing how local food systems differ in their ability to deliver low-cost nutrients around the year. Reducing and stabilizing the cost of acquiring a nutritious diet is important not only for those who buy all their food but also for farmers who use markets to complement what they grow. Purchased foods from local markets contribute substantially to the diets of agricultural households in Africa (*16*) and are especially important in lean seasons and for diet diversity beyond what can be produced and stored on the household’s own farm (*17*, *18*).

Our third major finding is that prices for animal-sourced foods had the least seasonality. This is one reason why the CoNA had less seasonality in Tanzania and Ethiopia than in Malawi, since their least-cost nutrient sources included more animal products. Overall, these findings point to opportunities for further improvement in low-cost, relatively stable supplies of animal-sourced foods, in addition to improvements in market access that would help people overcome seasonality in local production of plant-based foods.

Our analysis reveals the potential for high-frequency, high-density price observations to reveal the ability of local agroecosystems and food markets to deliver nutritionally complete diets at low cost, using data on food composition to compute the least-cost combination of foods that meet all essential nutrient requirements at each time and place. Protocols and software tools to automate the computation of least-cost diets allow us to extract nutrient costs from food price data over a total of 25,522 market-months, thereby measuring food system performance in ways that directly inform efforts to improve year-round access to nutritious diets in both rural and urban areas. Future studies may apply this method to identify the causes of differences in seasonality including local agricultural calendars, trade opportunities, and storage costs interacting with consumer demand, affecting both peaks in diet costs that harm consumers and seasonal lows that affect farm income and farming-dependent populations.

One key limitation of these analyses is that governments may not collect prices for all foods that could be low-cost sources of essential nutrients, at the times and locations where they are needed by people at risk of malnutrition. Other limitations include variation in the nutrient composition of each food especially after cooking, variation in peoples’ nutrient requirements, and variation in retail prices within the month at each market, all of which are subject to further research. Last, our measure of seasonality in this paper is limited to harmonic fluctuations, which is just one component of all variation. Future work could address different kinds of price differences and identify ways to improve agricultural production, storage, and transport to stabilize diet costs and improve year-round affordability of nutritious diets.

## MATERIALS AND METHODS

### Data sources

The food prices used in this study are historical file data provided by national statistical services in each country. Prices were originally collected for the purpose of measuring inflation using a consumer price index, based on a list of all goods and services needed to represent national average per capita consumption in that country over an entire year. Since individuals can substitute foods seasonally, and observed diets may not actually meet their nutritional needs, to address the impact of climate fluctuations on cost of nutrients, we link food prices with the nutrient composition of each item and model the least-cost combination of foods needed to meet human requirements of each nutrient.

For Tanzania, the National Bureau of Statistics collected monthly retail food prices of 71 food and nonalcoholic beverage items from all 21 regions of mainland Tanzania between January 2011 and December 2015. Price data are collected from different types of outlets, including open markets, supermarkets, neighborhood shops, groceries, shopping centers, and other retail outlets. The monthly price surveys are conducted in urban regional headquarters in all 21 regions in approximately four outlets per item. For nonprocessed food items, price collectors go to the shops/markets on three consecutive days for price collection and retain the median of those three observations.

In Malawi, the National Statistical Office assembled monthly price data for 55 food items in 29 market locations across 25 administrative districts between January 2007 and December 2016. Unlike the Tanzania dataset, all 29 markets in this dataset are in rural towns, 17 of which are the district capitals known as “boma” markets, and the remaining 12 are in other towns. The data are collected during the first 2 weeks of each month usually from three retail shops preselected by the National Statistical Office or vendors subject to data collectors’ judgement. They retain the geometric mean of the three observations. To reduce the disproportional effect of extreme values on model results, we have winsorized outliers beyond the top 1% of all ratios between reported price and the median for each item, replacing those outliers with the cutpoint value for that item.

The Ethiopia prices were obtained from the Consumer Price Survey, collected by the Central Statistical Agency (CSA). Monthly retail food prices considered in this study cover 97 food items in 120 markets from 57 zones of 11 administrative regions between January 2002 and December 2016 of 15 years. Like the dataset in Tanzania, the surveys are conducted in towns and cities. To ensure the survey to be nationally representative, the CSA also assigns the number of markets in each region to be proportional to the region’s share of total urban population in Ethiopia. CSA enumerators collect three price quotations from traders, retailers, and consumers in the first 15 days of each month. They retain the median of those three prices and, before our receipt of the data, also trimmed outliers below the 1st and above the 99th percentile of each item.

After assembling each country’s archival price data, we converted their units of measure to local currency per kilogram of edible matter (LCU/kg), and matched the item’s description to entries in local food composition tables (*19*–*21*) where available. To fill gaps where no local composition data are available, we used the U.S. Department of Agriculture National Nutrient Database for Standard Reference (SR28) (*22*). For data visualization and analysis, we also converted food prices to LCU per 100 kcal and classified foods on the basis of an adjusted form of the Minimum Dietary Diversity for Women guidelines (*23*) into six major mutually exclusive food groups: (i) grains, white roots and tubers, and plantains (“starchy staples”); (ii) pulses, nuts, and seeds; (iii) dairy and eggs, meat, poultry, and fish (“animal foods”); (iv) fruits and vegetables; (v) oils and fats; and (vi) sweets. Last, we dropped food items that have nutrients but would not be included in substantial quantities for adult meal plans such as infant foods and condiments. There are finally 61, 48, and 82 food items included in the analysis for Tanzania, Malawi, and Ethiopia, respectively, representing all six major food groups. Descriptive statistics and numerical results are reported in the annex of extended data.

### Computation of least-cost diets

To identify the most affordable sources of all essential nutrients, we automate the computation of least-cost diets at every time and place using linear programming approaches that were originally formulated to solve this and related problems during the Second World War (*24*). With each food’s market price and nutrient composition as fixed parameters, we obtain the quantity of each food that delivers all nutrients within fixed lower and upper bounds at the lowest total cost. This least-cost diet for all nutrients is defined as the solution to

min{*C* = Σ*_i_p_i_* × *q_i_*}, subject to six kinds of constraint:

(i) Σ*_i_a_ij_* × *q_i_* ≥ EAR*_j_*.

(ii) Σ*_i_a_ij_* × *q*_i_ ≤ UL*_j_*.

(iii) Σ*_i_a_ij_* × *q_i_* ≤ AMDR_*j*,upper_ × *E*/*e_j_*.

(iv) Σ*_i_a_ij_* × *q*_i_ ≥ AMDR_*j*,lower_ × *E*/*e_j_*.

(v) Σ*_i_a_ie_* × *q_i_* = *E*.

(vi) *q*_1_ ≥ 0, *q*_2_ ≥ 0, *q*_3_ ≥ 0,…, *q_i_* ≥ 0.

The objective is lowest diet cost given the price of each food (*p_i_*), choosing quantities (*q_i_*) to meet or exceed the population’s estimated average requirement (EAR) for nutrient *j* given the quantity of nutrient *j* in each food *n_ij_*, within the further constraint of overall estimated energy needs (*E*), while remaining below upper levels (UL) for most micronutrients and the chronic disease risk reduction (CDRR) upper bound for sodium, and within a range for macronutrients determined by acceptable macronutrient distribution ranges (AMDR_lower_ and AMDR_upper_) as percentages of daily energy needs (*E*). The reference number *e_j_* is the energy density of macronutrients, which is 4 kcal per gram of protein and carbohydrate and 9 kcal per gram of lipid. In the analysis, we included 21 nutrients, including 3 macronutrients (protein, fat, and carbohydrate), 8 minerals (calcium, iron, magnesium, phosphorus, zinc, copper, selenium, and sodium), and 10 vitamins (vitamin C, thiamin, riboflavin, niacin, vitamin B6, folate, vitamin B12, vitamin A, retinol, and vitamin E). Using this same framework, we also computed the CoCA for daily subsistence, using only starchy staples to meet the constraint of energy needs alone.

All the dietary reference intakes applied in our analysis include the most updated EAR, UL, AMDR, and estimated energy requirement developed by the U.S. Institute of Medicine (*25*), and we used healthy, not pregnant and lactating women of 57 kg and 163 cm between 19 and 30 years old with low active physical activity level as the reference population group. EAR is the amount of nutrient intake value meeting the requirement of half healthy population. For nutrients other than sodium, the upper limit indicates the UL, which is the highest level of daily nutrient intake that is likely to pose no risk of adverse health effects for the general population; for sodium, we used the CDRR developed in 2019 as the UL considering the beneficial effect of reducing sodium intake on cardiovascular disease risk, hypertension risk, systolic blood pressure, and diastolic blood pressure (*26*). The AMDR provides a range of intakes for macronutrients that is associated with reduced risk of chronic disease.

To automate computations, we call the lpSolve package in R (*27*) to return solutions for each location every month. Those computations are done in nominal local currency terms to reflect choices at each place and time. Then, for comparison over time and across countries, we converted each diet cost into constant USD using 2011 PPP exchange rates provided by the World Bank (*28*). Since local inflation occurs from month to month but PPP conversion factors are reported for each calendar year, we smooth over 12 months using the least squares technique as implemented in Stata using the -denton- command (*29*).

### Measurement of seasonality

We extracted the magnitude and timing of seasonal fluctuations using harmonic regression, also known as a trigonometric model. This approach uses sine and cosine functions over time, offering a parsimonious representation using just two parameters to estimate smooth, symmetric rise and fall of a variable. The harmonic approach has been shown to be more efficient than traditional monthly indicator models that estimate one coefficient for each month, and the harmonic form offers a closer fit for many seasonal patterns than other functional forms (*11*). The model specification is shown below

$$\text{ln}(C_{\mathrm{kt}})=\beta_{0}+\beta_{s}\times \text{sin}(2\pi \omega t)+\beta_{c}\times \text{cos}(2\pi \omega t)+\beta_{T}\times T(t)+\beta_{y}\times Y_{t}$$ (1)

where *C_kt_* is the monthly time series of food price or diet cost, in market *k* at month *t*. Coefficients of sin and cos terms, β*_s_* and β*_c_*, measure the magnitude (*A*) and peak timing (*P*) of seasonality where ω is a constant equal to ^1^/_12_, indicating 12 months per annual cycle. *T*(*t*) is a cubic polynomial term of *t*, controlling the trend of time series. *Y_t_* controls the fixed effect of crop years. In Tanzania and Malawi, the first month of a crop year is May, while it is October in Ethiopia (*30*).

Seasonal intensity is defined as the difference between annual peak and nadir prices normalized to a unit-free percentage of the nadir price, expressed as exp{(2*A*) − 1}, where *A* is the amplitude of the seasonality. Therefore, the seasonality is comparable across different food items, price indicators, and countries, and over time. The estimates of amplitude (*A*) and peak timing (*P*) and their variances are calculated using the δ method and the equations below (*4*)

$$A=\delta \sqrt{\beta_{s}^{2}+\beta_{c}^{2}},\text{where}\;\delta =1,\text{if}\;\beta_{c}>0,\text{and}\;\delta =-1,\text{if}\;\beta_{c}<0\;\text{and}$$ (2)

$$\text{Var}(A)=(\sigma_{s}^{2}\beta_{s}^{2}+\sigma_{c}^{2}\beta_{c}^{2}+2\sigma_{\mathrm{sc}}\beta_{s}\beta_{c})/(\beta_{s}^{2}+\beta_{c}^{2})$$ (3)

$$P=\frac{12(1-\frac{\varphi }{\pi })}{2},\text{where}\;\varphi =-\text{arctan}\;(\beta_{s}/\beta_{c})$$ (4)

$$\text{Var}(\varphi )=(\sigma_{s}^{2}\beta_{c}^{2}+\sigma_{c}^{2}\beta_{s}^{2}+2\sigma_{\mathrm{sc}}\beta_{s}\beta_{c})/{\left(\beta_{s}^{2}+\beta_{c}^{2}\right)}^{2}$$ (5)

where σ*_s_*, σ*_c_*, and σ*_sc_* are the SDs of β*_s_* and β*_c_* parameters, and their joint covariance. We also calculated the 95% CIs for *A* and *P* using a standard constant from a t-distribution of 1.96. The 95% CI of the amplitude is from $A-1.96\sqrt{\text{Var}(A)}$ to $A+1.96\sqrt{\text{Var}(A)}$. The harmonic regression models allow for assessing the significance of seasonal components, e.g., the significance of β*_s_* and/or β*_c_* parameters (for sin and cos terms, respectively). Thus, the peak timing estimates can be formally compared. If the 95% CI does not contain the value of zero, then seasonality will be determined significant (*31*).

For the seasonality analysis of energy intake compositions and cost components of CoNA contributed by different food groups, we applied a different harmonic model specification

$$I_{\mathrm{kt}}=\beta_{0}+\beta_{s}\times \text{sin}(2\pi \omega t)+\beta_{c}\times \text{cos}(2\pi \omega t)+\beta_{T}\times T(t)+\beta_{y}\times Y_{t}$$ (6)

where *I_kt_* is the energy intake compositions of CoNA in kilocalories and the cost components of CoNA from each food group in *k* market and time *t*. In this analysis, seasonal intensity is defined as the average absolute difference between the peak and nadir values in a yearly cycle, or simply the double of amplitude, 2*A*, estimated from Eq. 6.

We compared results from both harmonic regression and traditional monthly indicator models, for diet costs and individual food items across three countries, and the comparison results are shown in figs. S1 to S4. The model specification for the harmonic model followed Eq. 6 above, and the specification for the indicator variable approach is shown in Eqs. 7 and 8 below

$$I_{\mathrm{kt}}=\beta_{0}+\Sigma_{m}\beta_{m}\times M_{m}+\beta_{T}\times T(t)$$ (7)

$$\text{ln}(C_{\mathrm{kt}})=\beta_{0}+\Sigma_{m}\beta_{m}\times M_{m}+\beta_{T}\times T(t)$$ (8)

where *I_kt_* is the diet costs or food prices in the *k*th market at month *t*. *M_m_* is the dummy variable for calendar months, and we selected November as the base month in the analyses.

We used a multivariate mixed-effects model in estimations where observations are from multiple markets, with random intercepts and coefficients on the seasonal terms (sin and cos terms) by markets. If an estimation was based on observations from a single market, an ordinary least squares model was applied instead. All regression models were run in Stata/SE 15.1.

## Supplementary Material

http://advances.sciencemag.org/cgi/content/full/6/49/eabc2162/DC1

Adobe PDF - abc2162_SM.pdf

Seasonality of diet costs reveals food system performance in East Africa

## SUPPLEMENTARY MATERIALS

Supplementary material for this article is available at http://advances.sciencemag.org/cgi/content/full/6/49/eabc2162/DC1

## Appendix

View/request a protocol for this paper from *Bio-protocol*.
