# Supplementary material for: Seasonality of diet costs reveals food system performance in East Africa
Source: Sci Adv. 2020 Dec 4;6(49):eabc2162. doi: 10.1126/sciadv.abc2162 (PMC7821891; doi:10.1126/sciadv.abc2162)
Supplement: http://advances.sciencemag.org/cgi/content/full/6/49/eabc2162/DC1 [file supp_6_49_eabc2162__index.html]

Science Advances | Science AdvancesAAASSearchScience AdvancesMenu

## Supplementary Materials

# Seasonality of diet costs reveals food system performance in East Africa

Yan Bai, Elena N. Naumova, William A. Masters

Download Supplement

**This PDF file includes:**

- Figs. S1 to S4
- Tables S1 to S7

**Files in this Data Supplement:**

- Adobe PDF - abc2162\_SM.pdf
