# Supplementary material for: Seasonality of diet costs reveals food system performance in East Africa
Source: Sci Adv. 2020 Dec 4;6(49):eabc2162. doi: 10.1126/sciadv.abc2162 (PMC7821891; doi:10.1126/sciadv.abc2162)
Supplement: http://advances.sciencemag.org/cgi/content/full/6/49/eabc2162/DC1 [file supp_6_49_eabc2162__1.pdf]

Science Advances
AAAS

advances.sciencemag.org/cgi/content/full/6/49/eabc2162/DC1

Supplementary Materials for

Seasonality of diet costs reveals food system performance in East Africa

Yan Bai, Elena N. Naumova, William A. Masters*

*Corresponding author: Email: william.masters@tufts.edu

Published 4 December 2020, *Sci. Adv.* **6**, eabc2162 (2020)
DOI: 10.1126/sciadv.abc2162

This PDF file includes:

Figs. S1 to S4
Tables S1 to S7

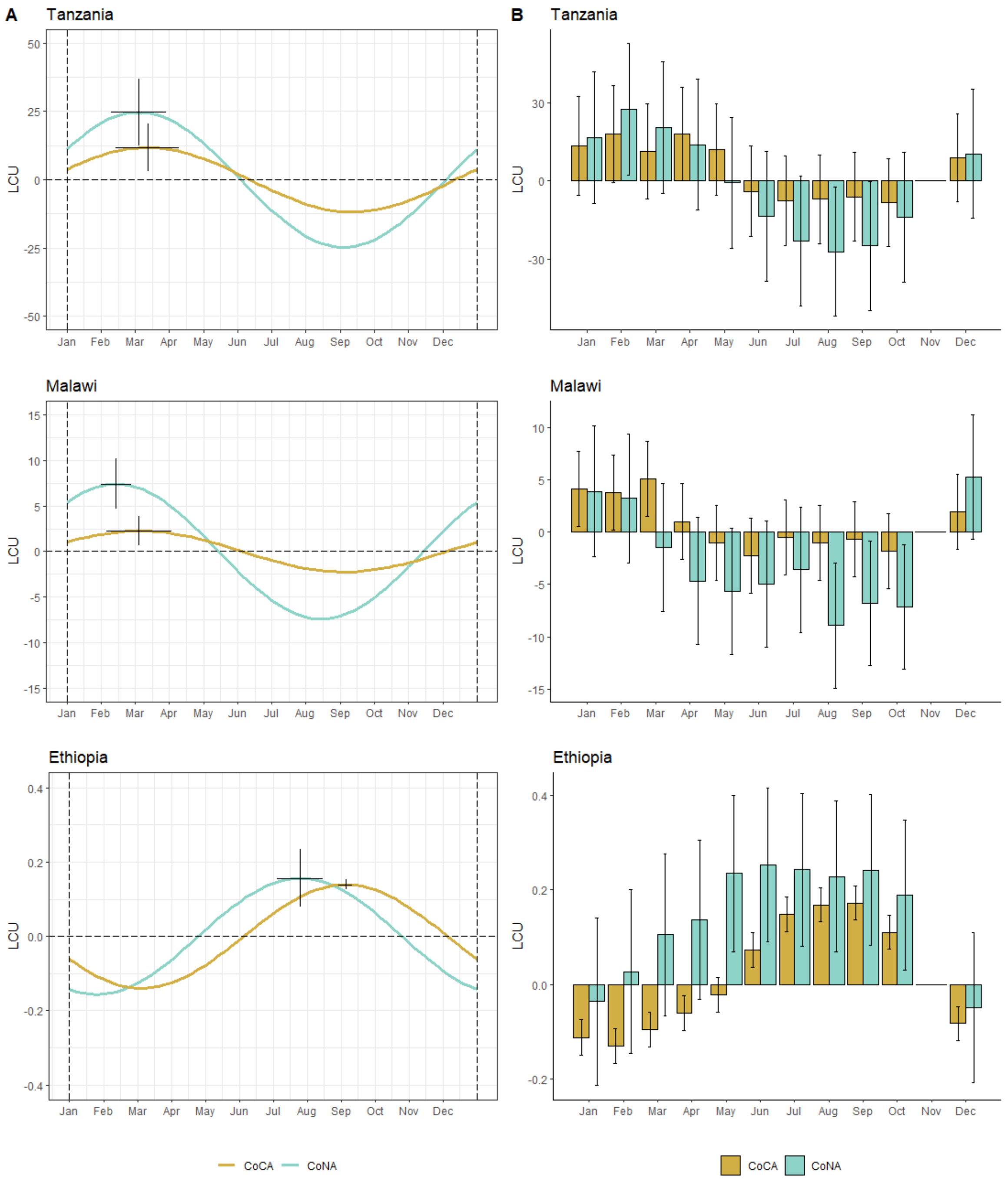

Fig. S1. Seasonality in diet costs for nutrient adequacy (CoNA) and caloric adequacy (CoCA): comparison between harmonic models and monthly indicators (LCU/day). (**A**) displays estimated harmonic seasonality over a one-year cycle, with error bars showing 95% confidence intervals around the magnitude of seasonal intensity along the vertical axis and peak month along the horizontal axis. (**B**) shows seasonal variation as differences by calendar month, using November as the base period for end of the dry season in Tanzania and Malawi, and end of rainy season in Ethiopia, with error bars showing 95% confidence intervals around monthly differences in LCU/day.

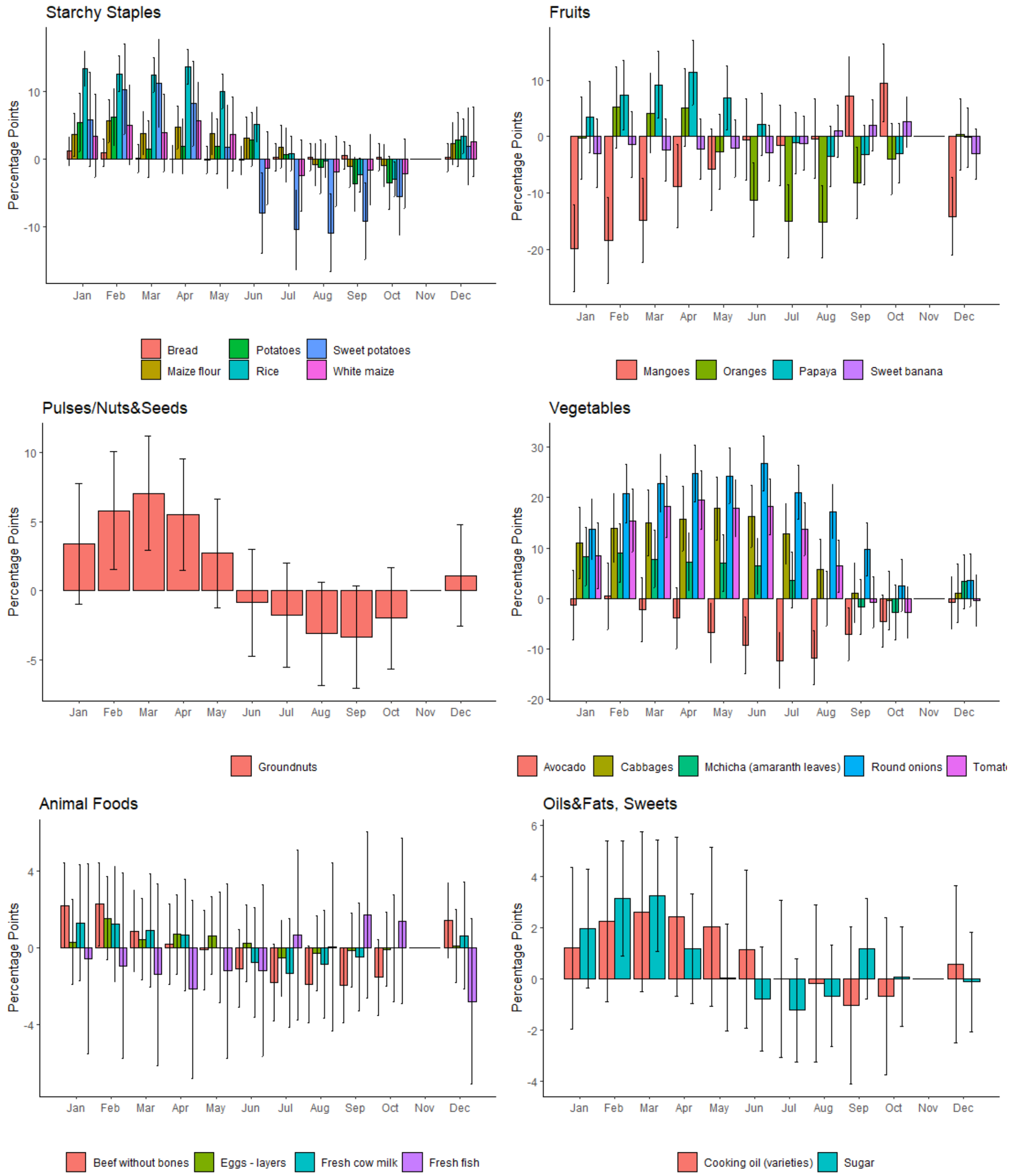

Fig. S2. Seasonality in retail prices of common food items in Tanzania. Data shown are percent differences by calendar month for selected items in each food group, relative to its price in November. Error bars show 95% confidence intervals.

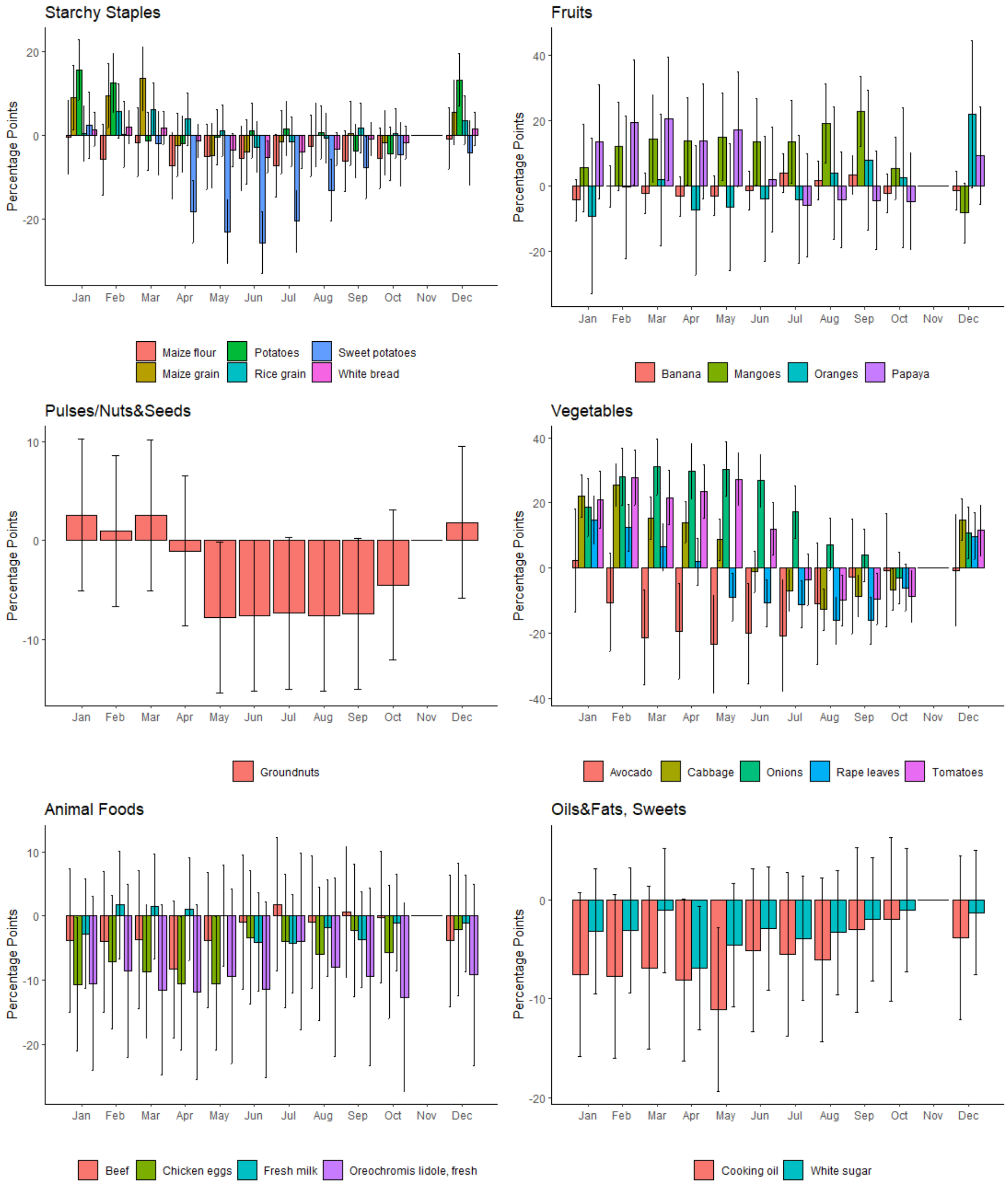

Fig. S3. Seasonality in retail prices of common food items in Malawi. Data shown are percent differences by calendar month for selected items in each food group, relative to its price in November. Error bars show 95% confidence intervals.

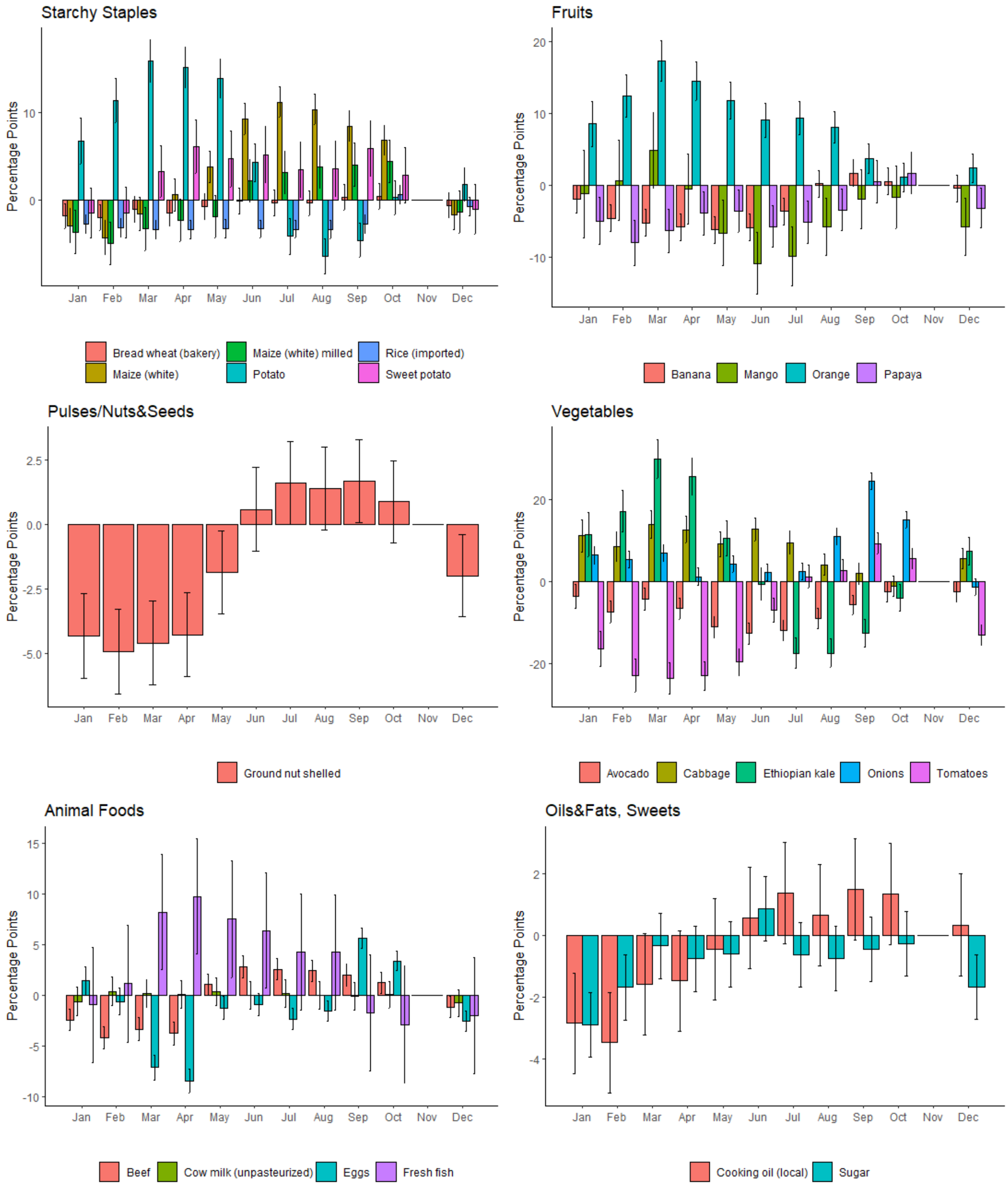

Fig. S4. Seasonality in retail prices of common food items in Ethiopia. Data shown are percent differences by calendar month for selected items in each food group, relative to its price in November. Error bars show 95% confidence intervals.

Table S1. Descriptive statistics and seasonality for monthly prices of all foods in Tanzania between 2011-2015

Food Groups	No	Food Items	Food Prices (LCU per 100kcal) Obs.	Mean	SD	CV	Peak Time Estimate	95% CI		Seasonal Intensity Estimate	95% CI	
Starchy Staples	1	Biscuit	1,236	105.4	100.5	1.0	8.6	1.4	15.8	1.3%	(5.0%)	8.1%
	2	Bread	1,236	82.3	23.0	0.3	0.5	(3.3)	4.3	0.7%	(1.1%)	2.5%
	3	Bun	1,236	64.0	23.0	0.4	4.4	0.4	8.4	2.7%	(1.2%)	6.7%
	4	Cassava flour	1,063	44.3	20.3	0.5	10.4	6.9	13.9	2.5%	(0.5%)	5.6%
	5	Cassava fresh	1,236	45.6	16.2	0.4	1.1	0.3	1.8	10.1%	4.5%	16.0%
	6	Cooking bananas	1,236	97.3	43.2	0.4	11.6	5.6	17.6	2.2%	(2.3%)	6.9%
	7	Finger millet	1,236	46.8	13.3	0.3	4.8	(0.0)	9.7	1.6%	(1.0%)	4.3%
	8	Maize flour	1,236	27.8	5.2	0.2	3.0	2.1	3.9	6.0%	3.5%	8.5%
	9	Pastry	1,236	73.2	19.9	0.3	4.9	1.7	8.1	2.6%	(0.3%)	5.5%
	10	Potatoes	1,236	177.4	49.2	0.3	2.2	1.4	3.0	9.6%	5.3%	14.0%
	11	Rice	1,236	44.4	8.4	0.2	3.0	2.8	3.2	17.6%	15.0%	20.3%
	12	Spaghetti	1,236	37.5	3.8	0.1	11.8	9.4	14.1	0.9%	(0.2%)	1.9%
	13	Sweet potatoes	1,232	84.6	29.2	0.4	2.4	1.9	2.9	21.2%	13.4%	29.4%
	14	Wheat flour	1,236	36.4	3.7	0.1	2.5	1.6	3.3	2.7%	1.0%	4.4%
	15	White maize	1,236	18.3	6.1	0.3	2.5	1.4	3.6	6.5%	0.7%	12.6%
Pulses, Nuts and Seeds	16	Beans (soya)	1,236	41.6	7.4	0.2	2.5	2.1	3.0	9.5%	6.3%	12.8%
	17	Lentils	1,235	72.7	21.1	0.3	3.4	2.5	4.3	8.0%	4.3%	11.9%
	18	Natural groundnuts	1,236	38.8	7.0	0.2	2.8	2.0	3.7	8.8%	4.2%	13.5%
	19	Red dry beans	1,236	47.0	8.7	0.2	3.0	2.1	3.8	6.7%	3.9%	9.6%
Animal Foods	20	Beef Sausage	1,236	280.7	46.5	0.2	1.9	(1.1)	4.9	0.8%	(1.1%)	2.7%
	21	Beef with bones	1,236	555.1	94.0	0.2	1.3	0.8	1.8	6.3%	4.4%	8.3%
	22	Beef without bones	1,236	299.2	50.8	0.2	1.7	1.2	2.2	5.2%	3.1%	7.3%
	23	Dried fish	1,137	666.4	325.3	0.5	0.5	(0.9)	1.9	8.0%	(3.6%)	21.1%
	24	Dried sardines	1,236	255.4	138.9	0.5	3.3	2.0	4.5	6.3%	3.3%	9.3%
	25	Eggs - layers	1,188	478.2	66.0	0.1	2.7	0.6	4.8	1.3%	(0.6%)	3.3%
	26	Eggs - traditional	1,236	649.4	164.8	0.3	5.6	(3.0)	14.1	0.6%	(1.7%)	2.9%
	27	Fresh cow milk	1,236	174.8	39.2	0.2	1.6	0.1	3.1	2.3%	(0.5%)	5.1%
	28	Fresh fish	1,236	1226.5	379.0	0.3	9.1	6.9	11.3	3.0%	(1.0%)	7.3%
	29	Fried fish	1,193	874.6	341.2	0.4	1.1	(1.9)	4.0	2.7%	(3.0%)	8.8%
	30	Goat meat	1,231	229.9	47.3	0.2	1.1	0.0	2.2	2.4%	0.6%	4.3%
	31	Industrially bred chicken	981	523.7	108.1	0.2	0.8	(5.9)	7.4	0.5%	(1.9%)	2.9%
	32	Pork meat	1,234	132.9	30.5	0.2	1.0	(0.3)	2.3	2.0%	0.1%	4.1%
	33	Powdered milk	1,236	474.6	68.3	0.1	1.2	(3.0)	5.4	0.6%	(1.2%)	2.5%
	34	Traditionally bred chicken	1,236	755.3	194.1	0.3	0.2	(1.1)	1.4	3.4%	1.0%	5.8%
Fruits and Vegetables	35	Apples (imported)	1,216	1187.3	308.0	0.3	3.7	1.4	6.0	5.4%	(0.9%)	12.2%
	36	Avocado	1,236	112.8	51.4	0.5	1.1	(0.0)	2.3	12.3%	4.9%	20.2%
	37	Bitter tomatoes	1,236	598.4	270.5	0.5	2.4	0.4	4.3	4.0%	(1.6%)	10.1%
	38	Cabbages	1,236	302.7	166.1	0.6	4.0	3.4	4.7	20.9%	14.1%	28.1%
	39	Carrots	1,236	418.9	222.1	0.5	4.9	3.9	6.0	11.9%	6.8%	17.3%
	40	Coconut mature	1,236	757.7	290.1	0.4	11.5	10.7	12.4	11.3%	6.5%	16.2%
	41	Egg plant	1,236	583.5	249.9	0.4	5.9	(1.5)	13.2	1.7%	(4.1%)	7.9%
	42	Green bell pepper	1,236	1575.8	606.7	0.4	4.4	2.8	5.9	8.8%	3.0%	15.0%
	43	Green peas	1,236	808.6	283.0	0.4	3.6	2.0	5.1	9.1%	3.6%	14.9%
	44	Green pepper (hot)	1,236	1029.4	419.8	0.4	9.3	7.4	11.3	7.0%	1.7%	12.6%
	45	Ladies finger (okra)	1,236	713.9	255.6	0.4	11.8	7.8	15.9	2.7%	(3.2%)	9.1%
	46	Lemons	1,236	695.8	333.7	0.5	1.7	1.2	2.2	35.9%	24.6%	48.3%
	47	Limes	1,024	1120.6	709.7	0.6	0.7	(0.2)	1.6	21.1%	9.6%	33.7%
	48	Mangoes	1,226	247.1	106.9	0.4	8.2	7.8	8.7	29.7%	19.5%	40.8%
	49	Mchicha (amaranth leaves)	1,236	342.8	166.0	0.5	3.1	2.2	3.9	12.8%	7.5%	18.4%
	50	Oranges	1,230	257.7	89.1	0.4	2.0	1.2	2.8	15.1%	6.7%	24.2%
	51	Papaya	1,236	370.9	133.9	0.4	3.6	2.6	4.7	13.4%	7.1%	20.1%
	52	Pineapples	1,236	411.3	137.1	0.3	8.6	8.0	9.1	20.9%	13.5%	28.7%
	53	Round onions	1,236	382.1	115.0	0.3	4.9	4.4	5.4	26.7%	20.2%	33.6%
	54	Sweet banana	1,236	199.6	80.0	0.4	8.8	7.4	10.3	6.3%	0.4%	12.6%
	55	Tomatoes red	1,236	533.5	201.2	0.4	4.4	3.8	4.9	25.8%	18.7%	33.3%
Oils and Fats	56	Cooking fat	1,236	145.1	82.8	0.6	2.3	0.4	4.2	2.4%	(0.8%)	5.8%
	57	Cooking oil	1,236	75.4	51.0	0.7	3.2	1.8	4.7	3.4%	1.0%	5.9%
	58	Margarine	1,236	99.8	25.2	0.3	4.3	0.8	7.8	1.4%	(0.3%)	3.2%
Sweets	59	Honey	1,236	151.9	56.1	0.4	1.3	(0.9)	3.5	1.9%	(1.4%)	5.4%
	60	Mixed fruits jam	1,236	278.3	52.4	0.2	1.8	(1.0)	4.6	1.0%	(1.3%)	3.3%
	61	Sugar	1,236	51.5	5.1	0.1	2.0	0.9	3.1	2.3%	0.4%	4.2%

Note: Peak time shows as a continuous variable from 0-12, where 0 indicates the start and 12 as the end of a calendar year.

Table S2. Descriptive statistics and seasonality for monthly prices of all foods in Malawi between 2007 to 2016

Food Groups	No	Food Items	Food Prices (LCU per 100kcal)				Peak Time			Seasonal Intensity		
			Obs.	Mean	SD	CV	Estimate	95% CI		Estimate	95% CI	
Starchy Staples	1	Biscuits	1,301	12.7	6.4	0.5	2.9	(0.4)	6.2	4.1%	(3.1%)	11.8%
	2	Cassava	2,522	7.1	6.3	0.9	8.0	(6.2)	22.2	0.9%	(6.6%)	9.0%
	3	Maize flour	2,791	7.6	5.5	0.7	0.9	(0.8)	2.5	6.4%	(1.1%)	14.5%
	4	Maize four (whole grain)	1,178	9.0	4.6	0.5	11.9	10.5	13.2	10.8%	2.4%	20.0%
	5	Maize grain	3,419	3.0	2.6	0.9	1.6	0.6	2.6	10.7%	2.9%	19.1%
	6	Maize grain, Admarc	3,048	2.2	1.7	0.8	3.4	1.4	5.3	6.4%	1.2%	11.8%
	7	Mandazi (fried-dough)	1,297	17.8	16.9	1.0	11.2	9.6	12.8	13.3%	4.0%	23.4%
	8	Potatoes	3,380	26.1	21.8	0.8	1.4	0.9	1.9	22.6%	14.5%	31.2%
	9	Rice grain	3,039	8.6	5.8	0.7	3.0	(1.1)	7.0	2.4%	(3.8%)	9.0%
	10	Sweet potatoes	2,897	12.7	10.9	0.9	11.8	11.2	12.4	25.2%	16.2%	35.0%
	11	White bread	3,340	10.8	5.0	0.5	1.1	0.2	2.0	5.3%	1.5%	9.2%
Pulses, Nuts and Seeds	12	Brown beans	3,380	12.8	8.4	0.7	0.6	(0.2)	1.4	13.0%	6.7%	19.7%
	13	Cowpeas	1,025	13.9	7.1	0.5	1.9	1.1	2.7	20.6%	8.9%	33.5%
	14	Groundnuts	3,030	8.4	6.1	0.7	1.2	(0.1)	2.4	9.0%	1.1%	17.5%
	15	Pigeon peas	730	15.1	7.9	0.5	1.9	(0.3)	4.2	6.5%	(4.5%)	18.6%
	16	White beans	3,033	11.6	7.5	0.6	1.1	(0.1)	2.2	8.8%	2.0%	16.0%
Animal Foods	17	Beef	3,248	34.0	22.5	0.7	9.4	5.4	13.4	4.2%	(2.9%)	11.9%
	18	Chicken	1,838	67.9	50.2	0.7	2.6	0.9	4.3	8.8%	(1.5%)	20.2%
	19	Chicken eggs	3,187	65.7	41.8	0.6	9.9	7.1	12.7	6.8%	(0.1%)	14.3%
	20	Cichlid, dried	2,736	280.1	199.5	0.7	9.4	7.5	11.3	8.3%	1.2%	15.8%
	21	Fresh milk	2,828	54.1	70.4	1.3	2.6	(0.5)	5.7	3.3%	(4.0%)	11.1%
	22	Goat	3,276	74.3	44.4	0.6	9.3	7.9	10.7	9.1%	3.0%	15.5%
	23	Oreochromis lidole, dry	1,366	316.1	251.8	0.8	10.1	6.4	13.8	5.1%	(1.3%)	11.9%
	24	Oreochromis lidole, fresh	499	141.4	139.1	1.0	9.7	7.1	12.3	8.4%	(0.4%)	18.0%
	25	Pork	1,538	35.0	16.7	0.5	8.7	5.3	12.0	5.5%	(5.1%)	17.3%
	26	Powdered milk	2,034	27.2	20.9	0.8	11.0	8.3	13.8	11.3%	0.9%	22.6%
	27	Sardine	3,406	91.3	65.6	0.7	6.8	6.0	7.5	15.4%	6.0%	25.7%
Fruits and Vegetables	28	Avocado	663	20.5	12.4	0.6	10.7	9.7	11.6	32.8%	17.7%	49.9%
	29	Banana	3,115	24.5	19.6	0.8	8.1	6.9	9.3	6.3%	(0.0%)	13.1%
	30	Cabbage	3,221	37.5	26.9	0.7	2.1	1.9	2.3	46.9%	37.5%	57.0%
	31	Chinese cabbage	1,263	151.4	102.5	0.7	1.5	0.9	2.2	33.0%	17.7%	50.2%
	32	Cucumber	644	177.4	98.0	0.6	9.5	8.7	10.2	52.7%	23.9%	88.1%
	33	Eggplant	1,018	107.0	56.5	0.5	1.6	0.7	2.4	19.2%	5.9%	34.2%
	34	Green beans	894	155.9	98.9	0.6	2.9	1.1	4.8	15.2%	(4.9%)	39.5%
	35	Guava	415	30.2	16.6	0.6	9.3	7.7	10.9	25.1%	7.2%	46.1%
	36	Mangoes	1,258	19.4	16.0	0.8	6.4	5.5	7.3	38.2%	17.3%	62.9%
	37	Okra	2,823	78.1	77.9	1.0	9.4	8.8	10.0	28.7%	16.9%	41.6%
	38	Onions	3,424	97.8	79.6	0.8	3.8	3.3	4.2	41.8%	32.1%	52.1%
	39	Oranges	546	66.1	50.5	0.8	11.3	9.6	13.0	24.0%	5.7%	45.6%
	40	Papaya	468	67.3	50.6	0.8	2.6	1.7	3.5	32.8%	12.2%	57.2%
	41	Pumpkin	406	67.9	63.5	0.9	11.8	10.4	13.2	52.0%	17.8%	96.1%
	42	Pumpkin leaves	2,986	196.9	162.3	0.8	9.2	8.6	9.9	20.7%	10.7%	31.6%
	43	Rape leaves	3,258	46.0	37.0	0.8	1.5	1.1	2.0	32.7%	22.2%	44.2%
	44	Tomatoes	3,432	119.5	99.2	0.8	2.7	2.3	3.1	60.3%	46.1%	75.9%
Oils and Fats	45	Cooking oil	3,030	12.4	9.4	0.8	9.8	7.5	12.1	6.2%	(0.7%)	13.5%
	46	Cooking oil, refilled	3,202	7.7	4.7	0.6	9.7	7.9	11.6	8.3%	1.6%	15.4%
Sweets	47	Brown sugar	3,392	8.0	5.7	0.7	8.6	6.4	10.9	4.5%	(1.2%)	10.6%
	48	White sugar	2,903	6.9	4.7	0.7	11.6	8.5	14.7	4.1%	(0.3%)	8.8%

Note: Peak time shows as a continuous variable from 0-12, where 0 indicates the start and 12 as the end of a calendar year.

Table S3. Descriptive statistics and seasonality for monthly prices of all foods in Ethiopia between 2002 to 2016

Food Groups	No	Food Items	Food Prices (LCU per 100kcal)				Peak Time			Seasonal Intensity (%)		
			Obs.	Mean	SD	CV	Estimate	95% CI		Estimate	95% CI	
Starchy Staples	1	African millet	11,269	0.2	0.1	0.7	8.4	7.9	8.9	11.1%	9.0%	13.4%
	2	Barley black	12,423	0.2	0.1	0.6	8.2	7.8	8.7	8.8%	7.0%	10.6%
	3	Barley white	17,169	0.2	0.1	0.6	8.3	8.0	8.7	10.0%	8.6%	11.5%
	4	Barley white milled	1,033	0.4	0.3	0.6	11.8	8.2	15.3	1.8%	(2.8%)	6.7%
	5	Bread wheat (bakery)	19,749	0.4	0.2	0.5	7.8	6.6	9.0	1.6%	0.5%	2.7%
	6	Durrah	8,071	0.1	0.1	0.7	8.3	7.8	8.7	17.8%	14.6%	21.1%
	7	Hulled barley	3,747	0.2	0.2	0.7	9.1	8.2	10.0	6.7%	4.0%	9.5%
	8	Kocho (unprocessed)	8,271	0.3	0.3	1.3	11.2	6.9	15.4	1.3%	(1.8%)	4.4%
	9	Maize (white)	19,180	0.1	0.1	0.6	7.3	7.1	7.5	16.2%	13.9%	18.5%
	10	Maize (white) milled	6,849	0.2	0.1	0.6	7.8	7.3	8.2	9.1%	7.0%	11.3%
	11	Oats	4,886	0.2	0.2	0.8	8.4	7.5	9.3	7.0%	4.5%	9.6%
	12	Oats milled	9,544	0.4	0.3	0.7	9.5	7.4	11.5	1.2%	0.4%	2.0%
	13	Potatoes	20,162	0.6	0.4	0.7	3.5	3.3	3.8	23.0%	19.8%	26.3%
	14	Rice (imported)	18,032	0.3	0.2	0.5	7.6	7.2	8.0	1.9%	1.4%	2.3%
	15	Sorghum milled	959	0.2	0.1	0.6	9.7	5.4	14.0	9.2%	0.1%	19.1%
	16	Sorghum red	13,867	0.1	0.1	0.6	8.7	8.5	9.0	16.6%	13.7%	19.6%
	17	Sorghum white	13,531	0.1	0.1	0.7	8.6	8.3	8.9	13.9%	11.8%	16.1%
	18	Spaghetti (local)	17,840	0.5	0.2	0.5	7.4	6.9	7.9	2.3%	1.5%	3.1%
	19	Sweet potatoes	10,985	0.3	0.2	0.9	6.3	5.8	6.9	8.3%	4.5%	12.3%
	20	Teff black (red) milled	1,697	0.2	0.2	0.7	8.6	7.7	9.5	6.1%	3.2%	9.0%
	21	Teff mixed milled	2,685	0.3	0.2	0.6	7.9	7.2	8.6	5.2%	3.1%	7.3%
	22	Teff white milled	2,590	0.3	0.2	0.6	8.3	7.6	9.0	4.4%	2.5%	6.4%
	23	Wheat black (red)	12,893	0.2	0.1	0.6	8.4	8.1	8.8	9.9%	8.2%	11.6%
	24	Wheat mixed milled	824	0.2	0.1	0.7	8.9	7.6	10.2	10.0%	5.7%	14.6%
	25	Wheat white	17,905	0.2	0.1	0.6	8.1	8.0	8.3	12.7%	11.3%	14.0%
	26	Wheat white milled	1,838	0.2	0.1	0.7	7.7	6.8	8.6	6.0%	2.7%	9.4%
Pulses, Nuts and Seeds	27	Chickpeas	19,275	0.2	0.2	0.7	10.0	9.7	10.4	7.1%	6.1%	8.1%
	28	Fenugreek seed	18,027	0.4	0.3	0.7	10.2	9.6	10.8	4.7%	3.7%	5.7%
	29	Ground nut shelled	14,075	0.3	0.2	0.7	8.2	7.8	8.5	7.7%	6.3%	9.0%
	30	Haricot beans	10,816	0.2	0.1	0.7	5.5	5.1	5.9	13.3%	10.3%	16.3%
	31	Horse beans	19,620	0.2	0.2	0.7	8.0	7.9	8.2	15.9%	14.4%	17.5%
	32	Lentils	18,589	0.4	0.3	0.8	7.5	7.2	7.7	8.7%	7.5%	10.0%
	33	Lima beans	6,277	0.2	0.2	1.3	6.0	5.7	6.3	17.6%	13.2%	22.1%
	34	Linseed red	18,124	0.3	0.2	0.8	8.1	7.7	8.4	8.8%	7.2%	10.5%
	35	Linseed white	3,726	0.4	0.4	0.9	7.4	6.7	8.0	7.2%	4.0%	10.5%
	36	Niger seed	14,939	0.3	0.2	0.8	8.0	7.6	8.4	7.8%	6.1%	9.6%
	37	Peas green (dry)	10,640	3.1	1.9	0.6	7.6	7.4	7.8	15.1%	13.3%	17.0%
	38	Peas split	16,427	0.4	0.2	0.7	6.6	6.3	6.9	4.6%	3.7%	5.5%
	39	Sesame seed	9,377	0.3	0.2	0.8	7.0	6.5	7.5	7.5%	4.8%	10.3%
	40	Soya beans	5,191	0.2	0.2	0.7	7.6	7.3	8.0	13.6%	10.5%	16.7%
	41	Sunflower seed	13,463	0.4	0.3	0.7	10.1	8.0	12.2	1.9%	0.3%	3.6%
	42	Vetches	11,125	0.2	0.2	0.7	10.9	10.7	11.1	16.3%	14.6%	18.0%
Animal Foods	43	Beef	18,312	5.4	4.1	0.8	7.8	7.6	8.0	7.8%	6.9%	8.6%
	44	Camel meat	1,599	1.9	1.7	0.9	8.4	6.7	10.1	4.5%	1.1%	8.1%
	45	Camel milk	2,108	1.1	0.8	0.8	3.5	2.0	5.0	6.3%	0.1%	12.8%
	46	Cheese cottage	9,761	1.5	1.3	0.9	1.9	(0.2)	4.1	2.3%	(0.2%)	4.9%

	47	Cow milk (unpasteurized)	17,120	1.0	0.7	0.7	4.6	1.8	7.4	0.9%	(0.3%)	2.1%
	48	Eggs	20,139	2.3	1.7	0.7	10.2	9.9	10.5	8.1%	6.7%	9.6%
	49	Fresh fish	2,352	3.5	3.0	0.9	4.9	4.2	5.6	12.3%	7.6%	17.1%
	50	Goat milk	675	1.6	1.1	0.7	8.9	(0.5)	18.2	1.5%	(3.6%)	7.0%
	51	Sardines (imported)	9,246	6.2	3.9	0.6	4.5	(13.8)	22.9	0.1%	(0.9%)	1.2%
	52	Yoghurt (traditional)	13,753	1.9	1.4	0.7	7.0	4.3	9.6	0.7%	(0.8%)	2.3%
**Fruits and Vegetables**	53	Avocado	11,877	1.0	0.7	0.7	11.9	11.4	12.4	9.0%	5.8%	12.3%
	54	Banana	19,208	1.2	1.0	0.8	10.7	10.2	11.1	8.6%	6.8%	10.5%
	55	Beet root	17,910	1.5	1.2	0.8	4.4	4.0	4.8	12.6%	10.1%	15.1%
	56	Cabbage	18,281	1.7	1.3	0.8	3.6	3.1	4.1	14.5%	10.4%	18.9%
	57	Cactus	927	1.1	1.1	1.0	1.4	0.9	2.0	55.5%	25.3%	92.9%
	58	Carrot	17,459	1.8	1.4	0.8	5.8	5.6	6.1	25.9%	21.8%	30.1%
	59	Cauliflower	1,143	17.9	14.4	0.8	4.2	0.3	8.2	4.9%	(3.9%)	14.4%
	60	Ethiopian kale	16,608	1.3	1.3	1.0	2.8	2.6	3.0	46.9%	38.0%	56.4%
	61	Garlics	19,322	2.5	2.2	0.9	6.5	6.2	6.7	22.4%	19.5%	25.4%
	62	Grapes	346	22.0	20.6	0.9	4.5	1.8	7.1	14.5%	(3.1%)	35.3%
	63	Green peas	4,071	4.3	3.0	0.7	0.7	(2.1)	3.4	3.0%	(1.3%)	7.5%
	64	Green pepper	19,113	4.3	3.4	0.8	3.5	3.2	3.8	27.3%	23.3%	31.5%
	65	Hot pepper (dried)	15,342	1.0	0.8	0.8	7.5	7.4	7.6	26.6%	24.3%	28.9%
	66	Leaks	5,677	2.0	1.7	0.9	10.5	9.4	11.7	8.0%	3.6%	12.5%
	67	Lemon	17,749	2.5	2.4	1.0	2.7	2.5	2.9	60.7%	52.1%	69.9%
	68	Lettuce	9,060	5.6	5.6	1.0	3.7	2.2	5.2	6.0%	0.9%	11.4%
	69	Mandarine	2,017	2.6	2.1	0.8	4.3	3.4	5.2	12.4%	6.2%	19.1%
	70	Mango	11,235	2.8	2.2	0.8	0.7	0.2	1.2	16.2%	11.1%	21.5%
	71	Onions	19,655	1.1	0.7	0.6	10.7	10.3	11.0	13.6%	11.6%	15.8%
	72	Orange	15,207	5.0	4.6	0.9	3.5	3.1	4.0	12.8%	9.5%	16.3%
	73	Papaya	12,767	2.0	2.1	1.0	8.8	8.0	9.6	6.2%	3.4%	9.1%
	74	Pumpkin	9,599	0.5	0.4	0.9	4.0	3.4	4.5	17.1%	12.6%	21.7%
	75	Spinach	9,859	1.3	1.2	0.9	2.1	1.5	2.8	15.7%	8.7%	23.1%
	76	Tomatoes	18,562	3.1	2.4	0.8	9.0	8.8	9.3	38.7%	31.7%	46.2%
**Oils and Fats**	77	Butter unrefined	18,461	1.0	0.7	0.7	4.8	4.5	5.1	12.1%	10.1%	14.0%
	78	Cooking oil (imported)	17,971	0.2	0.1	0.4	3.7	2.6	4.9	1.3%	0.8%	1.9%
	79	Cooking oil (local)	12,305	0.3	0.2	0.7	7.9	7.4	8.5	3.5%	2.4%	4.6%
	80	Vegetable butter (imported)	10,047	2.1	0.8	0.4	7.3	6.7	8.0	1.8%	1.0%	2.7%
**Sweets**	81	Honey	14,745	1.3	1.0	0.8	3.3	2.0	4.7	2.1%	1.0%	3.1%
	82	Sugar	18,651	0.3	0.1	0.5	6.4	6.1	6.7	3.0%	2.2%	3.8%

Note: Peak time shows as a continuous variable from 0-12, where 0 indicates the start and 12 as the end of a calendar year.

Table S4. Descriptive statistics and seasonality for national CoNA, CoCA, CoNA premium, energy composition and cost components of CoNA by food groups, and regional CoNA in Tanzania between 2011-2015

Categories	No	Items	Obs.	Mean	SD	CV	Peak Time			Seasonal Intensity		
							Estimate	95% CI		Estimate	95% CI	
**Indicators (LCU)**	1	CoNA	1,236	912.1	172.3	0.2	1.9	1.3	2.5	6.3%	3.7%	9.0%
	2	CoCA	1,236	378.4	122.0	0.3	2.4	1.2	3.5	5.9%	0.8%	11.3%
	3	CoNA premium	1,236	533.7	133.5	0.3	1.7	0.9	2.5	6.3%	2.4%	10.4%
**CoNA Energy Composition (kcal)**	1	Starchy Staples	1,236	1,317.8	78.6	0.1	10.3	4.6	15.9	6.1	(7.5)	19.7
	2	Pulses, Nuts and Seeds	1,236	618.9	146.2	0.2	4.0	(2.0)	10.0	8.2	(11.5)	28.0
	3	Animal Foods	1,236	153.8	115.6	0.8	6.0	(0.8)	12.8	4.6	(12.1)	21.4
	4	Fruits and Vegetables	1,236	13.0	15.6	1.2	8.0	5.7	10.3	2.6	(1.7)	7.0
	5	Oils and Fats	1,236	4.1	31.0	7.5	12.0	10.2	13.8	6.2	(2.1)	14.6
	6	Sweets	1,236	0.0	0.0	.	.	.	.	.	.	.
**CoNA and Cost Components (2011USD)**	1	Starchy Staples	1,236	0.5	0.1	0.3	2.3	1.3	3.3	0.03	0.01	0.05
	2	Pulses, Nuts and Seeds	1,236	0.4	0.1	0.3	2.8	1.8	3.8	0.03	0.01	0.05
	3	Animal Foods	1,236	0.6	0.2	0.4	0.9	(1.1)	2.9	0.02	(0.01)	0.06
	4	Fruits and Vegetables	1,236	0.1	0.0	0.8	3.6	0.9	6.3	0.01	(0.00)	0.02
	5	Oils and Fats	1,236	0.0	0.0	7.6	0.1	(1.8)	1.9	0.00	(0.00)	0.01
	6	Sweets	1,236	0.0	0.0	.	.	.	.	.	.	.
	7	CoNA	1,236	1.5	0.2	0.2	2.2	1.5	2.9	0.08	0.05	0.12
**Regional CoNA (LCU)**	1	Dodoma	60	947.6	142.7	0.2	1.4	0.2	2.6	12.9%	(0.2%)	27.7%
	2	Kilimanjaro	60	1048.9	171.9	0.2	6.9	5.3	8.4	4.6%	(0.7%)	10.2%
	3	Arusha	60	1062.2	182.7	0.2	2.9	2.1	3.8	9.4%	5.0%	13.9%
	4	Tanga	60	865.4	111.1	0.1	3.2	1.7	4.7	6.1%	1.5%	10.9%
	5	Morogoro	60	1051.9	117.1	0.1	4.3	3.4	5.2	9.1%	6.2%	12.1%
	6	Pwani	60	945.8	73.4	0.1	2.3	1.3	3.3	7.1%	2.0%	12.3%
	7	Dsm	60	1145.2	164.0	0.1	9.7	1.6	17.9	0.9%	(1.9%)	3.8%
	8	Lindi	60	1036.2	155.5	0.2	2.6	1.9	3.3	8.0%	4.5%	11.6%
	9	Mtwara	60	921.0	126.4	0.1	4.1	2.2	5.9	8.0%	2.7%	13.6%
	10	Ruvuma	60	788.1	109.7	0.1	2.6	0.7	4.5	5.7%	(1.1%)	13.1%
	11	Iringa	60	880.8	137.0	0.2	1.9	1.2	2.5	13.5%	6.0%	21.4%
	12	Mbeya	60	852.9	83.4	0.1	1.5	0.7	2.3	7.6%	2.3%	13.2%
	13	Singida	60	932.5	194.9	0.2	1.1	0.2	1.9	24.7%	8.2%	43.7%
	14	Tabora	60	891.3	118.2	0.1	2.6	1.9	3.2	9.5%	5.6%	13.5%
	15	Rukwa	60	765.5	118.2	0.2	0.9	(0.4)	2.3	6.7%	(0.1%)	13.9%
	16	Kigoma	60	654.5	68.3	0.1	1.1	0.5	1.6	18.2%	9.9%	27.2%
	17	Shinyanga	60	900.0	180.2	0.2	0.5	(0.8)	1.7	8.9%	1.9%	16.4%
	18	Kagera	60	796.0	109.2	0.1	0.3	(2.1)	2.6	3.6%	(1.3%)	8.8%
	19	Mwanza	60	908.8	77.1	0.1	1.8	0.1	3.5	2.4%	(0.8%)	5.8%
	20	Mara	48	830.0	103.7	0.1	12.0	8.6	15.3	3.8%	(2.5%)	10.6%
	21	Manyara	48	912.7	78.6	0.1	3.3	1.0	5.6	4.2%	(0.4%)	9.0%

Note: Peak time shows as a continuous variable from 0-12, where 0 indicates the start and 12 as the end of a calendar year.

Table S5. Descriptive statistics and seasonality for national CoNA, CoCA, CoNA premium, energy composition and cost components of CoNA by food groups, and regional CoNA in Malawi between 2007-2016

Categories	No	Items	Obs.	Mean	SD	CV	Peak Time			Seasonal Intensity		
							Estimate	95% CI		Estimate	95% CI	
Indicators (LCU)	1	CoNA	3,480	129.6	73.6	0.6	1.1	0.6	1.7	10.0%	5.7%	14.6%
	2	CoCA	3,480	41.6	30.6	0.7	1.5	0.4	2.6	8.0%	1.5%	14.9%
	3	CoNA premium	3,480	88.0	54.6	0.6	0.8	(0.1)	1.7	9.0%	2.7%	15.6%
CoNA Energy Composition (kcal)	1	Starchy Staples	3,480	1,387.9	261.2	0.2	8.2	7.2	9.2	55.6	19.8	91.4
	2	Pulses, Nuts and Seeds	3,480	372.5	261.6	0.7	7.8	5.9	9.7	34.6	(9.7)	79.0
	3	Animal Foods	3,480	115.4	173.0	1.5	2.8	(0.1)	5.8	12.7	(7.6)	33.0
	4	Fruits and Vegetables	3,480	89.9	127.1	1.4	2.0	1.3	2.7	39.8	21.0	58.7
	5	Oils and Fats	3,480	132.5	150.3	1.1	1.5	0.1	3.0	24.7	(0.4)	49.8
	6	Sweets	3,480	9.4	78.3	8.3	2.4	1.2	3.6	14.7	3.1	26.4
CoNA and Cost Components (2011USD)	1	Starchy Staples	3,480	0.4	0.2	0.5	1.3	(0.5)	3.1	0.02	(0.01)	0.04
	2	Pulses, Nuts and Seeds	3,480	0.2	0.1	0.6	10.9	8.0	13.8	0.02	(0.00)	0.03
	3	Animal Foods	3,480	0.3	0.2	0.5	4.4	2.0	6.8	0.02	0.00	0.03
	4	Fruits and Vegetables	3,480	0.2	0.2	1.0	1.1	0.6	1.6	0.08	0.05	0.11
	5	Oils and Fats	3,480	0.1	0.1	1.1	1.5	0.2	2.8	0.02	0.00	0.04
	6	Sweets	3,480	0.0	0.0	13.4	2.2	0.2	4.1	0.00	(0.00)	0.01
	7	CoNA	3,480	1.2	0.4	0.3	1.3	0.8	1.8	0.13	0.08	0.17
Regional CoNA (LCU)	1	Balaka	120	134.1	91.8	0.7	0.8	(0.1)	1.7	22.4%	6.6%	40.6%
	2	Karonga	240	142.5	75.4	0.5	2.8	1.5	4.1	14.0%	2.2%	27.2%
	3	Mulanje	120	132.1	73.8	0.6	1.7	0.7	2.6	19.9%	3.9%	38.3%
	4	Chitipa	120	125.6	73.9	0.6	10.7	7.0	14.4	7.2%	(1.4%)	16.6%
	5	Dedza	120	121.6	71.0	0.6	5.1	(1.1)	11.3	7.0%	(7.7%)	24.0%
	6	Mzimba	240	145.9	81.3	0.6	2.4	0.9	3.9	14.0%	4.7%	24.2%
	7	Zomba	120	129.2	73.8	0.6	1.9	(0.5)	4.2	9.1%	(7.7%)	28.8%
	8	Kasungu	120	95.1	46.7	0.5	10.0	7.7	12.3	28.4%	4.9%	57.1%
	9	Machinga	120	143.6	86.5	0.6	6.4	(3.9)	16.6	2.0%	(10.1%)	15.8%
	10	Blantyre	120	105.5	59.4	0.6	0.7	(0.1)	1.5	28.5%	11.1%	48.6%
	11	Mangochi	120	129.2	73.8	0.6	1.9	(0.5)	4.2	9.1%	(7.7%)	28.8%
	12	Chiradzulu	120	113.5	67.4	0.6	1.0	(0.2)	2.2	11.8%	0.6%	24.2%
	13	Mchinji	120	96.1	43.9	0.5	2.6	0.4	4.9	14.2%	(5.2%)	37.7%
	14	Lilongwe	360	119.4	58.3	0.5	0.3	(1.0)	1.5	17.1%	4.3%	31.5%
	15	Dowa	120	138.7	64.9	0.5	11.7	10.5	12.8	35.2%	15.5%	58.2%
	16	Mwanza	120	134.5	72.6	0.5	1.5	0.6	2.4	13.0%	2.7%	24.3%
	17	Chikwawa	120	138.5	79.2	0.6	1.3	0.4	2.1	16.9%	4.9%	30.2%
	18	Nkhata Bay	120	142.1	83.4	0.6	3.6	(1.4)	8.7	4.7%	(4.5%)	14.8%
	19	Nkhotakota	120	147.6	72.8	0.5	0.9	(1.0)	2.7	15.6%	(5.5%)	41.4%
	20	Nsanje	120	124.7	72.7	0.6	0.8	(0.7)	2.3	7.8%	(1.0%)	17.5%
	21	Ntcheu	120	131.5	85.0	0.7	0.7	(0.4)	1.9	24.8%	4.0%	49.7%
	22	Phalombe	120	134.3	80.7	0.6	2.8	1.6	3.9	16.2%	5.0%	28.7%
	23	Rumphi	120	141.1	79.1	0.6	3.1	(1.4)	7.5	4.4%	(5.4%)	15.1%
	24	Salima	120	133.2	73.9	0.6	6.0	3.2	8.8	17.0%	(7.0%)	47.2%
	25	Thyolo	120	131.3	63.2	0.5	0.4	(1.1)	2.0	12.3%	0.4%	25.7%

Note: Peak time shows as a continuous variable from 0-12, where 0 indicates the start and 12 as the end of a calendar year.

Table S6. Descriptive statistics and seasonality for national CoNA, CoCA, CoNA premium, energy composition and cost components of CoNA by food groups, and regional CoNA in Ethiopia between 2002-2016

Categories	No	Items	Obs.	Mean	SD	CV	Peak Time			Seasonal Intensity		
							Estimate	95% CI		Estimate	95% CI	
Indicators (LCU)	1	CoNA	20,704	6.7	5.1	0.8	6.6	6.1	7.2	4.0%	2.5%	5.5%
	2	CoCA	20,806	1.9	1.2	0.6	7.9	7.7	8.1	13.9%	12.2%	15.6%
	3	CoNA premium	20,704	4.8	4.3	0.9	3.6	2.9	4.3	5.3%	3.8%	6.8%
CoNA Energy Composition (kcal)	1	Starchy Staples	20,704	1,088.7	371.7	0.3	3.3	2.5	4.2	50.9	31.6	70.2
	2	Pulses, Nuts and Seeds	20,704	521.8	387.9	0.7	9.3	6.1	12.4	14.6	(5.3)	34.6
	3	Animal Foods	20,704	277.0	131.7	0.5	7.2	5.8	8.5	8.1	0.7	15.5
	4	Fruits and Vegetables	20,704	120.4	128.5	1.1	9.8	8.7	11.0	15.8	9.2	22.5
	5	Oils and Fats	20,704	99.1	152.1	1.5	9.8	8.8	10.8	18.1	11.7	24.4
	6	Sweets	20,704	0.7	14.7	22.7	6.7	4.6	8.7	0.5	(0.4)	1.4
CoNA and Cost Components (2011USD)	1	Starchy Staples	20,704	0.23	0.11	0.5	7.1	6.2	7.9	0.01	0.01	0.02
	2	Pulses, Nuts and Seeds	20,704	0.21	0.14	0.7	6.3	5.1	7.4	0.01	0.00	0.02
	3	Animal Foods	20,704	0.67	0.27	0.4	6.3	4.4	8.1	0.01	(0.00)	0.02
	4	Fruits and Vegetables	20,704	0.17	0.26	1.5	5.9	5.1	6.7	0.03	0.01	0.04
	5	Oils and Fats	20,704	0.06	0.13	2.2	9.7	8.4	11.1	0.01	0.01	0.02
	6	Sweets	20,704	0.00	0.01	23.2	6.4	4.4	8.4	0.00	(0.00)	0.00
	7	CoNA	20,704	1.34	0.48	0.4	6.6	6.0	7.2	0.06	0.03	0.08
Regional CoNA (LCU)	1	North Western Tigray	360	6.6	4.0	0.6	8.7	7.3	10.1	7.4%	3.3%	11.7%
	2	Central Tigray	358	6.1	3.6	0.6	9.1	7.8	10.4	6.4%	3.6%	9.2%
	3	Eastern Tigray	357	6.7	4.8	0.7	8.4	6.2	10.6	5.4%	0.2%	10.9%
	4	South Tigray	360	6.9	5.8	0.8	9.3	7.6	10.9	9.4%	(0.3%)	19.9%
	5	Awsi (Zone 1)	360	7.4	4.7	0.6	9.6	7.3	12.0	5.5%	1.5%	9.5%
	6	Gabi (Zone 3)	360	7.3	4.8	0.7	9.0	7.1	10.9	4.3%	1.4%	7.3%
	7	North Gonder	359	6.2	3.7	0.6	6.7	1.6	11.8	2.0%	(6.4%)	11.2%
	8	South Gonder	360	5.2	3.0	0.6	7.5	6.1	8.8	9.1%	1.2%	17.6%
	9	North Wollo	360	5.9	3.7	0.6	10.3	6.1	14.6	2.5%	(1.6%)	6.7%
	10	South Wollo	359	5.5	3.3	0.6	9.6	7.8	11.3	4.3%	1.8%	6.9%
	11	North Shewa (R3)	358	6.9	5.6	0.8	8.1	5.9	10.2	5.4%	(0.5%)	11.7%
	12	East Gojam	359	5.4	3.2	0.6	5.3	2.2	8.3	2.7%	(2.0%)	7.6%
	13	West Gojam	359	5.2	3.3	0.6	8.3	5.9	10.7	5.2%	(0.8%)	11.5%
	14	Wag Himra	330	7.4	4.5	0.6	8.2	6.5	9.9	9.9%	2.3%	18.1%
	15	Awi/Agew	360	5.4	3.4	0.6	8.9	6.1	11.7	4.7%	0.8%	8.7%
	16	Oromia	180	5.4	3.0	0.5	7.1	6.0	8.2	6.0%	0.8%	11.5%
	17	West Wellega	359	6.1	3.8	0.6	7.3	5.7	8.8	4.7%	(0.6%)	10.2%
	18	East Wellega	360	6.3	4.2	0.7	5.2	3.7	6.8	11.7%	3.2%	20.9%
	19	Ilubabor	360	5.6	3.8	0.7	6.7	5.5	7.9	6.6%	0.0%	13.6%
	20	Jimma	360	5.8	4.0	0.7	3.7	(0.1)	7.5	2.1%	(0.5%)	4.9%
	21	West Shewa	358	5.9	3.5	0.6	0.8	(1.1)	2.7	5.2%	(0.3%)	10.9%
	22	North Shewa (R4)	356	7.6	5.9	0.8	7.7	5.4	9.9	6.6%	(2.4%)	16.3%
	23	East Shewa	360	6.0	3.6	0.6	6.3	4.7	7.8	3.1%	(0.9%)	7.4%
	24	Arsi	356	6.4	5.3	0.8	6.1	5.3	6.9	13.0%	3.0%	24.0%
	25	West Harerge	358	6.5	4.3	0.7	5.9	5.3	6.4	13.6%	7.7%	19.9%
	26	East Harerge	360	6.6	4.5	0.7	4.0	2.4	5.5	12.7%	6.4%	19.3%
	27	Bale	355	6.2	4.0	0.6	5.7	4.0	7.4	4.1%	(0.8%)	9.3%
	28	Borena	359	7.4	5.0	0.7	3.2	(0.9)	7.3	3.5%	(1.2%)	8.4%
	29	Siti	360	14.0	14.0	1.0	10.7	7.5	13.9	7.1%	(3.0%)	18.3%
	30	Fafan	360	8.4	5.3	0.6	2.9	1.0	4.8	6.7%	0.2%	13.6%

31	Liben	359	9.7	7.4	0.8	6.0	4.4	7.6	7.7%	(2.1%)	18.5%
32	Metekel	359	7.1	5.5	0.8	5.6	4.7	6.6	13.9%	4.8%	23.7%
33	Asosa	360	6.2	4.0	0.7	7.3	3.9	10.8	3.3%	(4.6%)	11.8%
34	Kemashi	355	6.8	4.6	0.7	4.7	4.0	5.3	25.2%	16.9%	34.1%
35	Gurage	359	6.3	4.1	0.7	6.7	2.8	10.6	1.9%	(4.1%)	8.2%
36	Hadiya	360	6.1	3.7	0.6	4.6	2.7	6.6	7.0%	(0.1%)	14.6%
37	Kembata Timbaro	359	7.8	6.2	0.8	6.6	2.9	10.2	2.5%	(5.1%)	10.6%
38	Sidama	356	6.1	3.9	0.6	5.9	4.7	7.1	7.2%	0.5%	14.3%
39	Gedio	360	7.5	5.5	0.7	0.1	(9.4)	9.6	0.8%	(4.6%)	6.6%
40	Wolayita	359	6.2	5.1	0.8	5.4	3.2	7.7	5.0%	(2.2%)	12.8%
41	South Omo	347	5.9	4.5	0.8	6.3	4.5	8.1	7.1%	(3.5%)	18.9%
42	Sheka	360	5.3	3.3	0.6	6.1	4.0	8.1	3.8%	(2.4%)	10.4%
43	Keffa	360	5.3	3.6	0.7	4.8	2.2	7.3	6.1%	(0.7%)	13.5%
44	Gamo Gofa	359	5.6	3.5	0.6	5.4	2.4	8.4	3.5%	(3.0%)	10.4%
45	Bench Maji	350	5.4	3.3	0.6	2.8	(4.7)	10.3	2.6%	(4.5%)	10.3%
46	Yem	177	8.5	6.2	0.7	5.5	4.9	6.0	36.7%	22.7%	52.3%
47	Segen Peoples'	717	6.5	4.8	0.8	6.4	5.5	7.2	10.2%	2.5%	18.4%
48	Dawro	350	7.8	5.4	0.7	3.7	2.0	5.5	10.7%	(2.9%)	26.3%
49	Basketo	180	6.3	5.4	0.9	6.1	4.9	7.3	13.8%	0.3%	29.1%
50	Konta	177	8.3	5.8	0.7	11.5	10.0	13.0	6.7%	0.2%	13.6%
51	Nuer	175	7.5	4.6	0.6	6.1	5.1	7.1	14.4%	2.4%	27.7%
52	Agnuak	222	8.1	9.1	1.1	6.2	5.2	7.2	27.7%	3.4%	57.8%
53	Majang	97	7.6	2.6	0.4	0.8	(1.1)	2.7	8.5%	(4.4%)	23.0%
54	Hareri	180	6.5	3.8	0.6	0.6	(26.2)	27.4	0.2%	(4.1%)	4.7%
55	Region 14	1,968	7.2	4.7	0.7	8.9	8.3	9.4	8.0%	5.8%	10.1%
56	Chirkos	179	6.1	4.2	0.7	8.5	7.5	9.5	11.9%	6.8%	17.3%
57	Dire Dawa	360	8.7	6.8	0.8	9.5	5.5	13.5	4.2%	(1.8%)	10.6%

Note: Peak time shows as a continuous variable from 0-12, where 0 indicates the start and 12 as the end of a calendar year.

Table S7. Example least-cost diets in Tanzania, Malawi and Ethiopia, by month (g/day)

Tanzania (Kigoma, 2015)

	Jan	Feb	Mar	Apr	May	Jun	Jul	Aug	Sep	Oct	Nov	Dec
White Maize grains	352	352	352	352	352	358	358	358	358	358	358	335
Cassava flour												23
Sweet Potatoes	59	59	59	59	59	64	64	64	64	64	64	69
Soybeans	172	172	172	172	172	164	164	164	164	164	164	168
Dried fish	17	17	17	17	17	17	17	17	17	17	17	17
Green pepper	18	18	18	18	18	14	14	14	14	14	14	10
Mchicha (amaranth leaves)						19	19	19	19	19	19	

Malawi (Kasungu, 2016)

	Jan	Feb	Mar	Apr	May	Jun	Jul	Aug	Sep	Oct	Nov	Dec
Maize grain	355	311	305	252	175	175	281	132	334	299	334	272
White bread	104	78	92	188	143	143		249	57	68	57	192
Sweet potatoes				69	2	2			51	67	51	
Brown beans							30					
Cowpeas	24	16	13									
Groundnuts									114	130	114	
Chicken eggs		232	225				356	405				
Powdered milk					164	164	58	61				
Beef	75			75								
Sardine									10	10	10	10
Mangoes					116	116						
Pumpkin	116		31									112
Rape leaves		85							84	3	84	
Avocado					35	35						
Cabbage				159								136
Guava	22		25				23	24		25		
Cooking oil	25	43	44	21	21	21	19	15				63
Cooking oil, refill				27								

Ethiopia (Mecha, West Gojam Zone, 2016)

	Jan	Feb	Mar	Apr	May	Jun	Jul	Aug	Sep	Oct	Nov	Dec
Maize (white)	244	254	292	279	269	309	176	175	326	175	185	295
Wheat white							172	141		135	67	
African millet	32	50	43	29	48	39		37	21	40	47	28
Sweet potato				16								
Chick peas	25	14		15	10							21
Horse beans	21	13			6							
Lima beans						47					41	
Ground nut shelled				36								
Cow milk (unpasteurized)	444	444	444	444	444	444	444	444	444	444	444	444
Sunflower seeds						18						
Ethiopian kale	35	2		12		30	54		7	4	17	7
Pumpkin	421	320	197	807	271	279			692	47	238	774
Spinach		58	137		90	14	132	146	96	123	38	30
Cooking oil (imported)	44	44	43		43	11	46	46	15	46	47	17

Note: The study as a whole uses a total of 25,522 least-cost diets, drawing from a potential list of 71, 55 and 97 distinct food items in Tanzania, Malawi and Ethiopia respectively. To illustrate the composition of an individual diet, and the degree of substitution among items and between food groups over the course of a year, we selected the one market location with the lowest average CoNA in each country, and show the composition of its least-cost diets each month over the most recent calendar year for which prices were available. This reveals which specific food items are selected each month at comparable locations in the three countries, in terms of grams per day. Of the three locations shown, the simplest example is from Tanzania, where people in the lowest-cost district (Kigoma) can meet their requirements most affordably by including 19 g/day of a green leafy vegetable (mchicha) during the June -November period, with a corresponding reduction in the need for green pepper and soybean.
